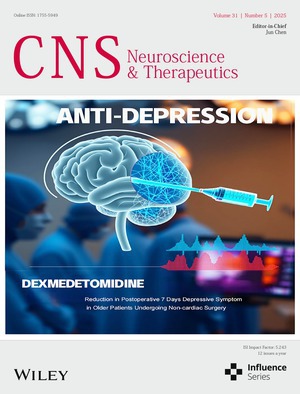# Front Cover

**DOI:** 10.1111/cns.70469

**Published:** 2025-06-23

## Abstract

The cover image is based on the article *Association of Dexmedetomidine With Postoperative Depressive Symptoms in Older Surgical Patients: A Prospective Multicenter Study* by Xinyu Hao et al., https://doi.org/10.1111/cns.70407.